# Hand washing practice among health care workers in Ethiopia: systemic review and meta-analysis, 2020

**DOI:** 10.1016/j.heliyon.2021.e06972

**Published:** 2021-05-08

**Authors:** Haileyesus Gedamu, Teshager W/giorgis, Getasew Tesfa, Yilkal Tafere, Minichil Genet

**Affiliations:** a Adult Health Nursing Department, Bahir Dar University, Bahir Dar, Ethiopia; b Pediatric Nursing Department, Bahir Dar University, Bahir Dar, Ethiopia; c Public Health Department, Debremarkos University, Debremarkos, Ethiopia; d Nursing Department, Bahir Dar Health Science College, Bahir Dar, Ethiopia

**Keywords:** Health care workers, Hand washing practice, Meta-analysis, Ethiopia

## Abstract

**Objective:**

Hand washing with soap and water is the single most weapon against infectious agents. Proper hand washing is not only reduces nosocomial infection, but also prevents the spread of current global concern Novel Corona viruses (COVID-19) and other viral illnesses like cold and flu. Therefore, the aim of this study is to assess hand washing practice among health care workers in Ethiopia.

**Methods:**

In the current meta-analysis, the target variables search from different databases, like Google Scholar, African Journals OnLine, PubMed, and Scopus. All necessary data extracted by using a standardized data extraction format. Heterogeneity across the studies was evaluated using the I^2^ index and Cochran's Q test. A random effect model computes to estimate the pooled proportion of hand washing practice among health care workers.

**Results:**

In this meta-analysis, we included fifteen observational studies summarize the proportional of hand washing practice among health care workers. In the current study, the pooled hand washing practices among Ethiopian was 57.87% (95% CI: 44.14–71.61). Subgroup analysis conduct to identifying the sources of heterogeneity.

**Conclusion:**

The overall pooled proportion of hand washing practice among health care workers was low. Hand washing with water and soap is recommended at least for 20 s to prevent contagious disease like Corona viruses.

## 1 Introduction

Hand washing is the process of mechanically removing soil and debris from the hands using plain water and soap [1, 2]. According to world health organization guidelines hand washing remains the most effective measure to prevent the mode of transmission of micro-organisms [3]. In the previous researcher reported that there are insufficient hand washing practices due to lack of appropriate equipment, inadequate knowledge and unfavorable attitudes among health care workers [4, 5, 6]. A study of medical students about hand washing practice showed that only 44.12% of the participants always washed their hands with soap and water [7]. Continuous health education improves hand washing practice among health care providers 69.9% [8].

According to Ethiopian national guidelines Hand hygiene is a general term referring to any action of cleansing hands [9]. Hand washing is effective measure in preventing infection transmission. Proportion of hand hygiene practice in different studies was 16.5%, 41% and 62.1% [10, 11, 12]. Only 87.5% health care providers used the hand hygiene products [13]. Lack of running water and soap in the wards is one of the major contributory that inhibited appropriate hand washing practice in health institution [14]. More than 70 % of U.S. and 66% of Canadian adults would avoid a health care facility or office if they found their restroom to be unclean [15]. Evidences showed that the proportion of hand wash before and after every patient contact was lowered 12.3% [16].

A study conducted in Australia among medical staff showed that about 67% of participants wash their hands before making hand contact with a patient [17]. In a study conducted in Nigeria the rate of hand washing before and after simple procedures were 13.6 and 59.7% respectively [18]. Nursing students had good hand washing practices (62.1%) as compared to medical students (19.6%) [19]. And also 70% of medical students used soap and water whereas only 6.36% used alcohol based agents for hand washing practice [20]. Scientific evidence showed that the average hand washing practice of midwives and nurses was 44.8% [21]. In Pakistan (96.10%) of participants had good hand washing practice [22]. Nurses had better (66%) hand wash practice than other professionals (63.8%) [23].

Many efforts were made to determine the level of hand washing practice among health care workers to prevent infectious diseases like the Novel corona virus. However, the proportion of hand washing practice among different study findings in Ethiopia was found to be inconsistent with discrepancy. Therefore, to address this gap, this systematic review and meta-analysis was conducted with the aim of evaluating the overall pooled proportion of the hand washing practice among health care workers.

## 2 Methods

### 2.1 Search strategy

This systemic review with meta-analysis conduct from the primary observational studies. The observational studies evaluate based on hand washing practice assessment materials (questionnaire). In terms of language, the articles published in English only were included in the analysis. The search was conducted in electronic databases, such as Google Scholar, African Journals OnLine, PubMed, and Scopus is using the following MeSH and free-text terms: hand washing practice’’, “hand hygiene practice”, “health care workers”, health care “professional” and “Ethiopia” (appendix 1). This searched was carried out from June 21 to July 27, 2020.

### 2.2 Inclusion and exclusion criteria

All original research articles were conducted only in Ethiopian settings that fulfill the following inclusion criteria included in this meta-analysis. Those articles which were published in English, conducted with cross-sectional study and having a quantitative research design. Those articles which didn't fully access at the time of our search process were excluded.

### 2.3 Data extraction

Data were extracted by three authors using a pre-piloted and standardized data extraction format prepared in a Microsoft excel. The data extraction sheet was piloted on five randomly selected papers and modified accordingly. This form includes the study characteristics, like author/s name, year of publication, study area, study design, sample size, practice, and the quality score of each study extracted from each included article by five independent authors. Any disagreements at the time of data abstraction were agreed upon by discussion and consensus.

### 2.4 Statistical analysis

The data extraction format includes primary author, publication year, region, study design, study hospitals, sample size, and proportion. After extraction, the data analyzed using STATA version 11 statistical software [24]. The pooled proportion of this meta-analysis reported as the pooled practice of health care workers about hand washing with 95% confidence intervals (CIs), p-values <0.05 were considered statistically significant. Heterogeneity across the studies evaluated using the I^2^ index and Cochran's Q test (I^2^ statistics below 25% indicated low heterogeneity, between 25% and 50% moderate heterogeneity, and over 75% high heterogeneity) [25]. Because the test statistic indicated significant heterogeneity among studies (I^2^ >75%, p < 0.05), a random effects model was used to evaluate practice of health care workers about hand washing practice with 95% confidence interval (CI).

To identify heterogeneity in the included studies, sub-group analysis by data collection techniques and study areas (both hospitals and health centers and hospitals only) intended to conduct hand washing practice among health care workers across the different regions in the country. Funnel plot asymmetry, Egger's and Begg-Mazumdar Rank correlation tests were used to check for publication bias. Two researchers independently carried out the statistical analysis and results from crosscheck for consistency.

## 3 Result

### 3.1 Study selection process

**Figure 1:**
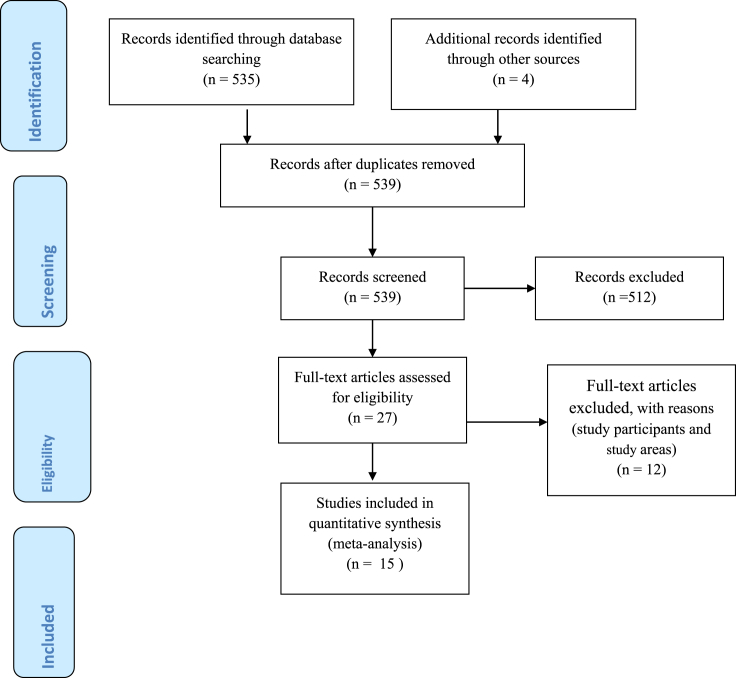
Flow chart to a selection of studies for a systematic review and meta-analysis of the proportion of hand washing practice among health care in Ethiopia, 2020.

The database search yields 539 articles retrieved from PubMed, Scopus, Google Scholar, and African Journals OnLine. Accordingly, 512 duplicate articles were removed. From the remaining 27 articles, 12 articles excluded because of their tittles and abstracts were not in line with our inclusion criteria (full article not found, different population, different setting and different outcome). Finally, 15 articles include for this systemic review and meta-analysis (Figure 1).

### 3.2 Characteristics of included studies

**Figure 2:**
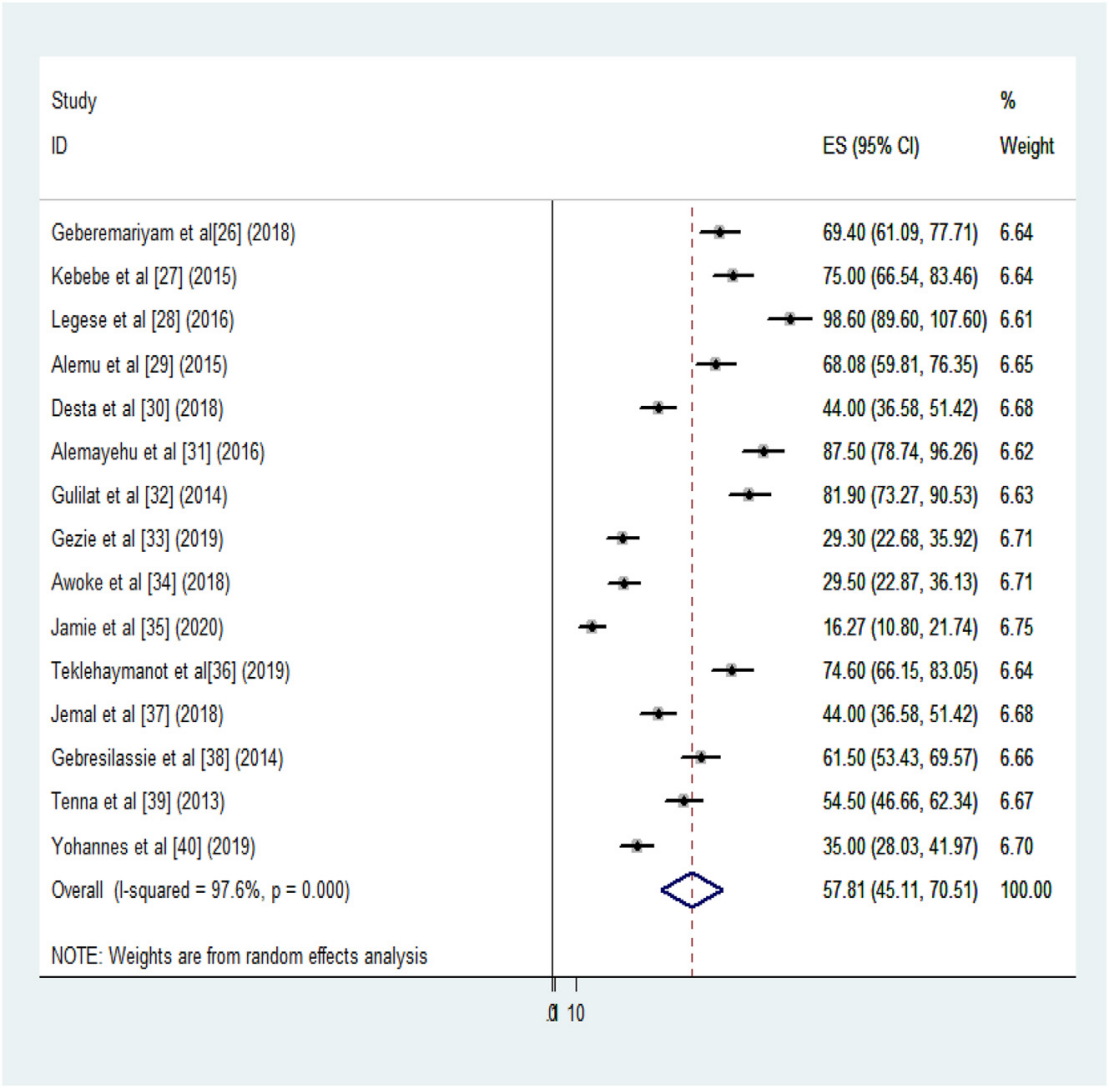
Meta-analysis (forest plot) of the prevalence on hand washing practice among health care workers in Ethiopia, 2020.

Fifteen studies with a total of 3198 health care workers include in our study. All included studies applied a cross sectional study design to evaluate hand washing practices of health care workers. The eligible fifteen studies were published from 2013 to 2020. The overall distribution of studies based on region included in this review were four from the Oromia region [26, 27, 28, 29], four from the Amhara region [30, 31, 32, 33], three from the Harar region [34, 35, 36] one from Afar [37] one from the Tigray [38] one from the Addis Ababa [39] and one from the Southern Nations Nationalities and People's Region (SNNPR) [40]. The highest level of hand washing practice was in the Oromia [28] and the lowest hand washing practice in the Harar region among health care workers [35]. Moreover, eleven studies conducted only in the hospitals and four studies in the hospitals and health centers (Table 1). In the case of data collection technique: nine studies used a self-administer questionnaire [26, 27, 28, 29, 30, 31, 33, 36, 39], two studies used both self-administer questionnaire and direct observation [34, 40] one structured interview and observational study [32] one only observational study [35] and one face to face interview, observation and focus group discussion [38] used for data collection technique. An individual who got training has good hand washing practice than an individual who didn't get [26, 30]. The low performance of hand washing was related to inadequate hand washing supply in the health facility [28, 35]. All of the studies reported high response rates (>90%). The overall percentage of hand washing practice among health care workers was 57.81% (95% CI: 45.11, 70.51) (Figure 2).

**Table 1:** Characteristics of primary studies included health care workers about hand washing practice in Ethiopia, 2020.

S. no	First Author	Publication Year	Region	Health Facility Name	Type of guideline	Sample Size	Level of hand washing practice % (95% CI)
1	Geberemariyam et al [26]	2018	Oromia	Arsi Hosp and HC	Local	648	69.40 (65.85,72.95)
2	Kebebe et al [27]	2015	Oromia	Jimma Hosp	WHO	17	75.00 (54.42,95.58)
3	Legese et al [28]	2016	Oromia	Agaro Hosp and HC	Local	73	98.60 (95.90,101.30)
4	Alemu et al [29]	2015	Oromia	Shenen Gibe Hosp	Local	47	68.08 (54.75,81.41)
5	Desta et al [30]	2018	Amhara	Debre Markose Hosp	CDC	150	44.00 (36.06,51.94)
6	Alemayehu et al [31]	2016	Amhara	Dessie Hosp	Local	208	87.50 (83.01,91.99)
7	Gulilat et al [32]	2014	Amhara	Bahir Dar Hosp &HC	Local	354	81.90 (77.89,85.91)
8	Gezie et al [33]	2019	Amhara	Dessie Hosp	National	191	29.30 (22.85,35.75)
9	Awoke et al [34]	2018	Harar	Harar Hosp	NM	110	29.50 (20.98,38.02)
10	Jamie et al [35]	2020	Harar	Harar Hosp	NM	166	16.27 (10.66,21.88)
11	Teklehaymanot et al [36]	2019	Harar	Harar Hosp	Local	125	74.60 (66.97,82.23)
12	Jemal et al [37]	2018	Afar	Dubti Hosp	NM	91	44.00 (33.80,54.20)
13	Gebresilassie et al [38]	2014	Tigray	Mekelle Hosp &HC	Local	483	61.50 (57.16,65.84)
14	Tenna et al [39]	2013	Addis Ababa	Addis Ababa Hosp	National	261	54.50 (48.46,60.54)
15	Yohannes et al [40]	2019	SNNPR	Hadya Hosp	National	274	35.00 (29.35,40.65)

Hosp = Hospital, HC = Health Center, NM = Not Mentioned, WHO = World Health Organization, CDC = Communicable Disease Control.

### 3.3 Pooled proportion of hand washing practice among health care workers in Ethiopia

**Figure 3:**
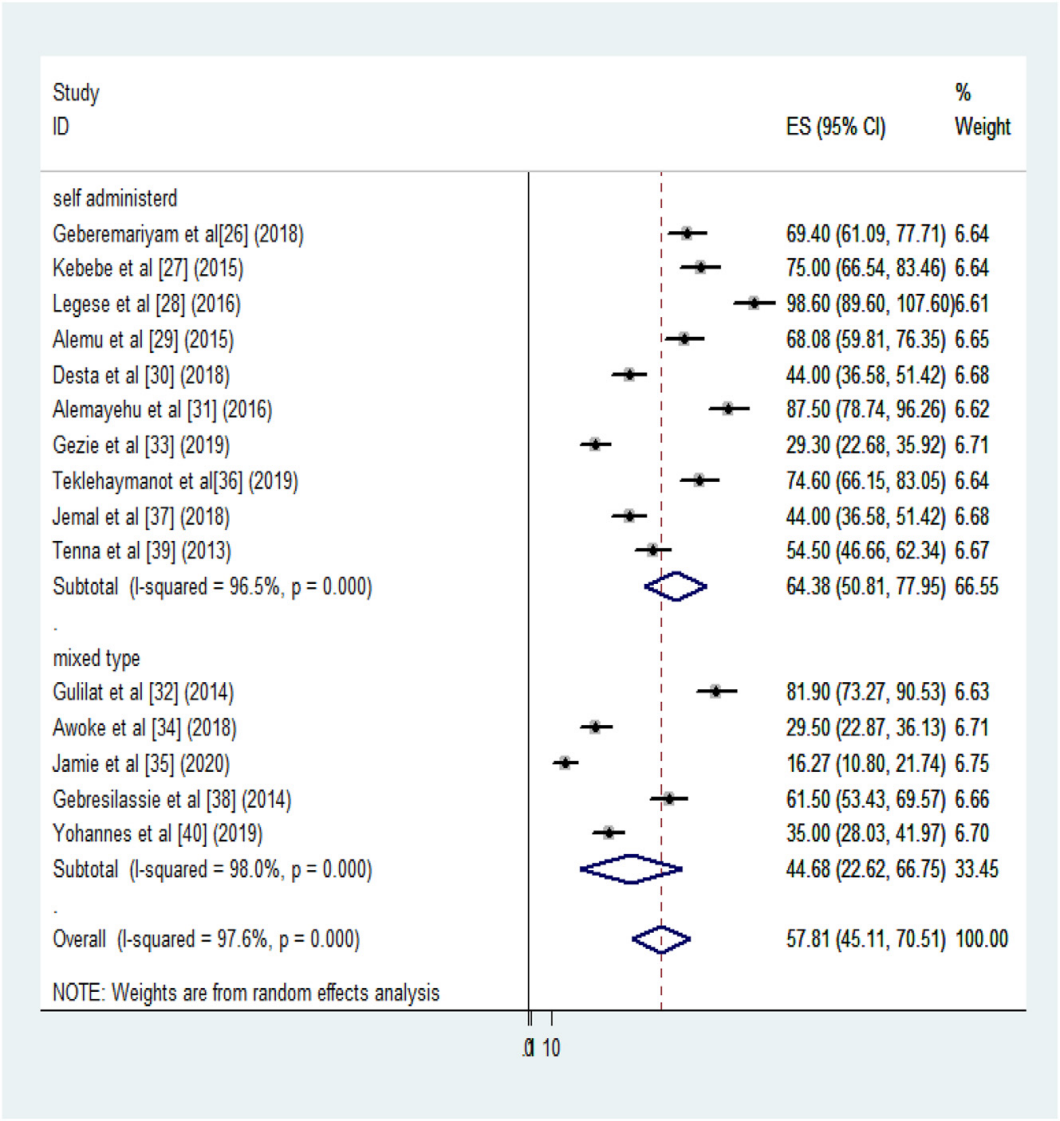
Forest plot of subgroup analysis by data collection technique on hand washing practices among health care workers in Ethiopia.

**Figure 4:**
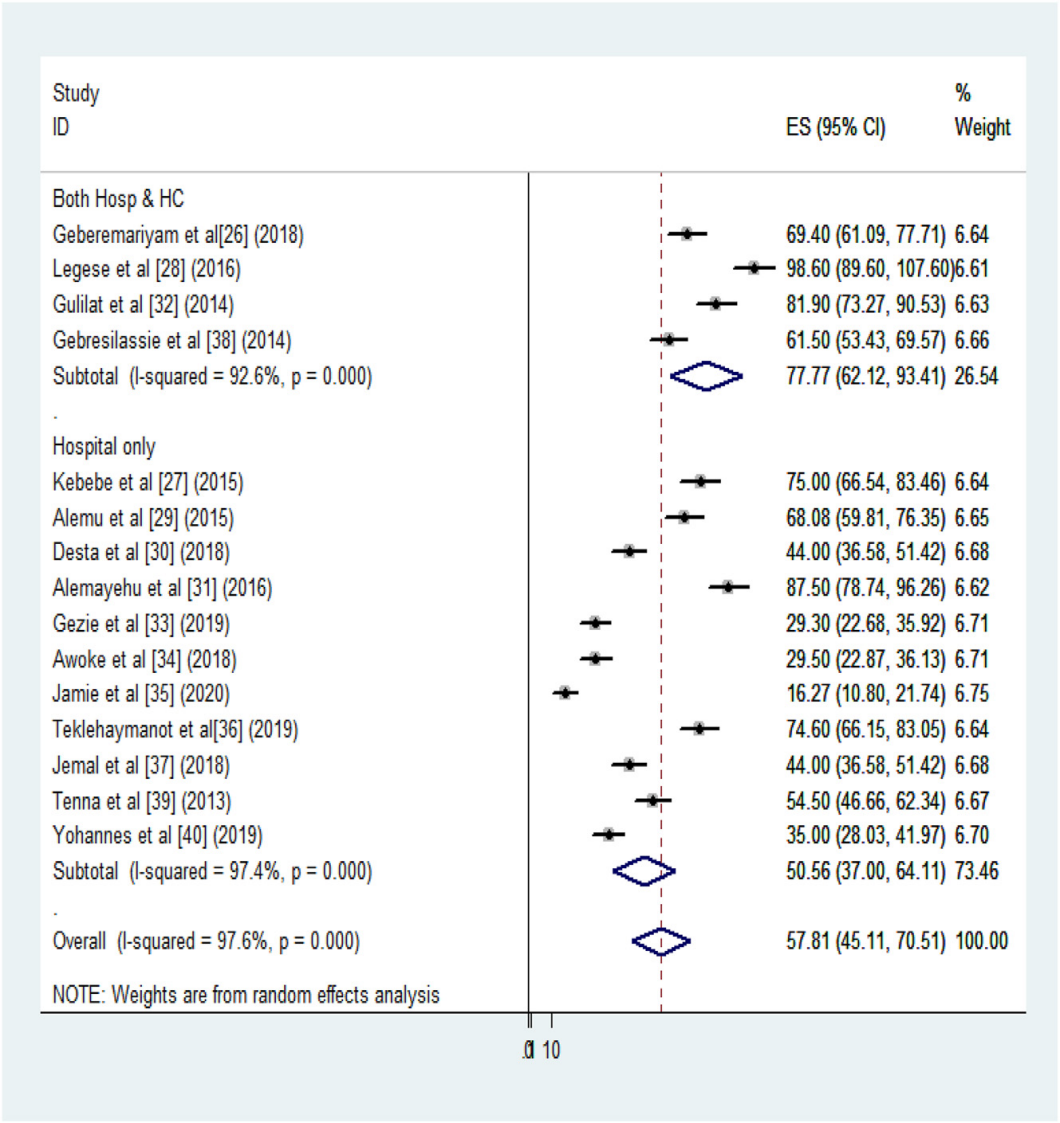
Forest plot of subgroup analysis by study area on hand washing practices among health care workers in Ethiopia.

In this study, the Pooled proportion of hand washing practice among health care workers in Ethiopia was 57.81% (95% CI: 45.11–70.51) (Figure 2). Using the random effects model statistically significant level of heterogeneity was observed in the included primary studies (I-squared = 97.6%; p < 0.001). Since there is heterogeneity in the included studies, we performed subgroup analysis. In order to identifying the sources of heterogeneity, we had conducted sub group analysis by using data collection technique (self-administer questionnaire only and mixed types) and study areas (both hospitals and health centers and hospitals only) to determine the pooled proportion of hand washing practice among health care workers (Figures 3 and 4). The result of subgroup analysis, revealed that, the low level of hand washing practice was observed among study groups used mixed (observational study and interview, self-administered) types of data collection technique of hand washing practices and health care workers who are working in hospitals only, which showed that 44.68% (95% CI: 22.62,66.75) and 50.56% (95% CI: 37.00,64.11) respectively.

### 3.4 Publication bias

**Figure 5:**
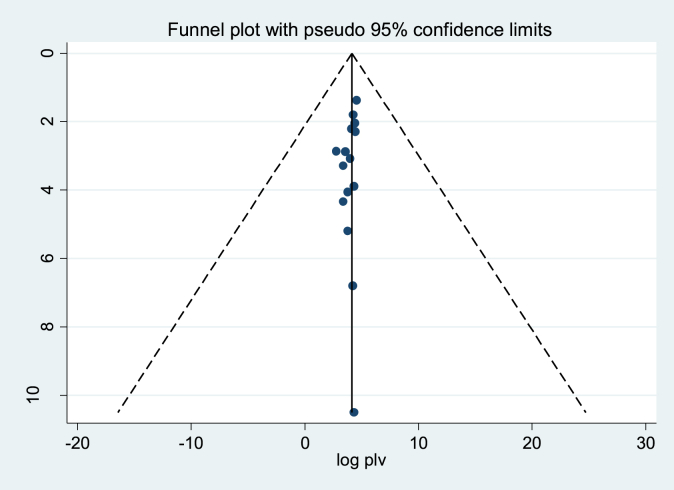
Meta funnels presentations of the proportion of hand washing practice among health care workers in Ethiopia, 2020, whereby SE PIV (standard error of proportion) plotted on the Y-axis and log PIV (logarithm of proportion) on the X-axis.

Both funnel plots of precision asymmetry and the Egger's test of the intercept were explore to identify the existence of publication bias in the included studies. Visual examination of the funnel plot showed symmetric distribution of studies implies no publication bias (Figure 5). We also conduct Egger's test of the intercept was -0.22 (95% CI: -0.44, 0.01), p > 0.05, this implies that there is no publication bias. Additionally we conducted sensitivity analysis, for the purpose of further investigating the potential source of heterogeneity observed in the hand washing practice among health care workers in Ethiopia. The result of sensitivity analyses using random effects model showed that there was no single study affected the overall hand washing practice of health care workers.

## 4 Discussion

In this study, systemic review and Meta-analysis was conducted to estimate the pooled proportion of the hand washing practice among health care workers in Ethiopia. The overall pooled proportion of the hand washing practice among health care workers was 57.81% (95% CI: 45.11–70.51). This finding is in line with those of studies conducted in Nigeria (55.2% and 59.7%) [18, 48], in India (62.1) [19] and in Rome (62.1) [12].

On the other hand, our finding suggested that the proportion of the hand washing practice was higher than studies conducted in Sudan (18.15) [49], in Japan (33.6%) [50], in Turkey (37%) [51], in Germany (41%) [11], in Syria (45.7%) [52], in Nepal (49.6%) [41] and Turkey (50%) [42]. The possible reason might be due to the study participants and methods of data collection. In the current review, the study participants were graduated health care workers, whereas, in some study area [41] both health care workers and students were incorporated. Also the methods of data collection in the current review were both self-administered questionnaire and interview, whereas in other study area [42] was only observational. However, this Meta-analysis was lower than studies conducted in several findings, 64.2% [45], 67% [17], 70% [20], 71% [53], 72.8% [46], 75% [43], 75.9% [54], 76% [44], 76.1% [55], 96.1% [22]. The possible explanation might be, the current review of hand washing practice among health care workers was pooled proportion results of fifteen studies, whereas, the others studies were conducted in a single area.

In the current study, sub-group analysis was done based on the study area and data collection technique used for conducted study. The result of the subgroup analysis showed that variability was observed in overall pooled hand washing practice among health care workers. Health care workers who are working in both hospitals and health centers had relatively highest pooled proportion of the hand washing practice than health care workers who are working in hospitals only with the results of 77.77 % and 50.56 % respectively. Due to the fact that, patient flow and work load is high in hospitals and they perceived that hand washing takes time as a result hand washing practice is low among health care workers who are working in hospitals [32, 47]. And also in Ethiopia context, physicians working in hospitals rather than in health centers and physicians have low hand washing practice when we compare from other health professionals particularly nurses [39, 41, 46].

In other words, studies conducted with self-administration questionnaires only of data collection technique showed that slight increment of hand washing practice (64.38%) as compared to studies conducted by mixed type of data collection techniques (44.68%). The possible reason might have, observational data collection technique reflects the hidden and actual activities of human beings, however, in self-administered questionnaire mostly reflects their knowledge, principles and obligation rather than their actual practice which is liable for social desire bias.

Although, there were some limitations, our study provided important information about the hand washing practice among health care workers in national level. Therefore, researchers and policy makers can easily compare on principle and the actual practice of hand washing practice among health care workers to prevent some nosocomial infection in the health facilities. However, the limitations are; first the present study was included only English articles were considered to provide this nationally based review. Second, the review didn't show the possible reason for the low hand washing practice among health care workers because of majority studies incorporated in this review were descriptive studies.

## Declarations

### Author contribution statement

Haileyesus Gedamu: Conceived and designed the experiments; Performed the experiments; Analyzed and interpreted the data; Wrote the paper.

Teshager W/giorgis: Performed the experiments; Wrote the paper.

Getasew Tesfa: Analyzed and interpreted the data.

Yilkal Tafere: Contributed reagents, materials, analysis tools or data; Wrote the paper.

Minichil Genet: Contributed reagents, materials, analysis tools or data.

### Funding statement

This research did not receive any specific grant from funding agencies in the public, commercial, or not-for-profit sectors.

### Data availability statement

Data included in article/supplementary material/referenced in article.

### Declaration of interests statement

The authors declare no conflict of interest.

### Additional information

No additional information is available for this paper.
